# Extracellular matrix composition of connective tissues: a systematic review and meta-analysis

**DOI:** 10.1038/s41598-019-46896-0

**Published:** 2019-07-22

**Authors:** Turney J. McKee, George Perlman, Martin Morris, Svetlana V. Komarova

**Affiliations:** 1 Shriners Hospital for Children – Canada, 1003 Décarie Boulevard, Montréal, Quebec H4A 0A9 Canada; 2 Schulich Library of Physical Sciences, Life Sciences and Engineering, McGill University, 809 rue Sherbrooke Ouest, Montréal, H3A 0C1 Canada; 3 Faculty of Dentistry, McGill University, 2001 Ave McGill College, Montréal, Quebec H3A 1G1 Canada

**Keywords:** Literature mining, Musculoskeletal system

## Abstract

The function of connective tissues depends on the physical and biochemical properties of their extracellular matrix (ECM), which are in turn dictated by ECM protein composition. With the primary objective of obtaining quantitative estimates for absolute and relative amounts of ECM proteins, we performed a systematic review of papers reporting protein composition of human connective tissues. Articles were included in meta-analysis if they contained absolute or relative quantification of proteins found in the ECM of human bone, adipose tissue, tendon, ligament, cartilage and skeletal muscle. We generated absolute quantitative estimates for collagen in articular cartilage, intervertebral disk (IVD), skeletal muscle, tendon, and adipose tissue. In addition, sulfated glycosaminoglycans were quantified in articular cartilage, tendon and skeletal muscle; total proteoglycans in IVD and articular cartilage, fibronectin in tendon, ligament and articular cartilage, and elastin in tendon and IVD cartilage. We identified significant increases in collagen content in the annulus fibrosus of degenerating IVD and osteoarthritic articular cartilage, and in elastin content in degenerating disc. In contrast, collagen content was decreased in the scoliotic IVD. Finally, we built quantitative whole-tissue component breakdowns. Quantitative estimates improve our understanding of composition of human connective tissues, providing insights into their function in physiology and pathology.

## Introduction

The ECM is a composite of cell-secreted molecules that offers biochemical and structural support to cells, tissues, and organs^1^. In humans, the composition of the ECM can be broadly summarized as a combination of water, protein, and polysaccharide, with the precise balance of these three compartments reflecting functional requirements of the tissues. The structural requirements determine the mechanical properties of the ECM, which depend on the protein composition of the matrix, particularly the abundance of collagen and elastin^1^. The physiological relevance of these properties extends beyond simple structural integrity. The cells surrounded by the ECM are capable of sensing its rigidity through integrin-mediated interactions with the matrix^2^. The mechanical properties of the matrix are then interpreted and affect motility, proliferation, differentiation, and apoptosis^3–6^. Thus, the knowledge of the precise composition of different tissues is important for understanding their structure-function relationship.

Connective tissue is one of the four basic types of human tissue, and is primarily composed of fibrous ECM components^7^. Tendons, ligaments, adipose tissue, bone, cartilage, and intervertebral disc (IVD) are connective tissues involved in diverse physiological roles including nutrient storage, endocrine function, and providing structural integrity^8–10^. The physical properties of connective tissues are similarly varied, perhaps best exemplified by contrasting adipose tissue, a loose tissue populated by large, lipid-containing adipocytes, with bone, a hard tissue consisting primarily of mineralized collagen fibrils^9,11^. The protein composition of human connective tissues is described as differing substantially, however quantitative and comparative studies on the subject are few and far between.

Connective tissue pathologies are often closely associated with alterations in the composition and structure of the ECM. This is particularly well studied for disorders that affect cartilage, such as osteoarthritis, and IVD degeneration. Osteoarthritis (OA), a common disorder occurring in 60% of adults over the age of 65 in Europe and North America, leads to pathological changes in the structure and composition of the cartilage matrix, which coincide with excessive ECM degradation^12^. Degeneration of the IVD is also linked to excessive matrix catabolism^13^. A great deal of variance exists around reported incidence rates of disc degeneration, however some reports suggest a rate as high as 70% in some populations, and the disorder is usually thought to be linked to back pain^14,15^. Adolescent idiopathic scoliosis occurs in approximately 2.5% of children aged 10–16, and was suggested to involve abnormalities in connective tissues^16^. It stands to reason that because of the matrix and tissue degradation observed in these disorders, there may be quantifiable differences between the protein composition of healthy and diseased connective tissues.

The goal of this study was to perform a systematic review and meta-analytic synthesis of the published literature reporting quantitative information on the protein composition of human connective tissues. The primary objective was to obtain estimates for absolute and relative amount of ECM proteins. We set two additional secondary objectives: 1) to quantify the changes in ECM composition in distinct pathologies; 2) to combine the outcomes for ECM proteins with reference values for other components to build quantitative estimates of whole tissue composition.

## Methods

### Information retrieval

A medical librarian (MM) constructed a search strategy (provided in Supplemental Materials and Methods) and performed a computerized bibliographic search of the Medline (OVID) database. This search strategy was then adapted to search EMBASE and SCOPUS. The searches were performed March 10, 2017 and returned 8,341 non-duplicate publications. The search of references and citations identified 7 additional publications. An updated search was performed on January 23, 2019 and returned an additional 1256 non-duplicate publications.

### Study selection

All screening and selection was performed by 2 co-authors (TJM and GP for the original search, TJM and SVK for the updated search). Abstract/title screening identified papers that contained information on ECM proteins derived from human tissue. During full text screening, the studies were selected if they contained 1) quantification of proteins; 2) the quantified proteins included those found in the ECM; 3) samples were taken from human bone, adipose tissue, tendon, ligament, cartilage or skeletal muscle; 4) the articles reported complete statistical information including sample sizes, mean values, and measures of spread (standard deviation or standard error). Exclusion criteria were 1) no other studies reported on the protein or tissue of interest, making synthesis impossible; 2) the reported units were incompatible with the transformation to μg protein/mg dry tissue. All conflicts that arose throughout the screening process were discussed by the two reviewers until consensus was reached.

### Data collection

The reported tissue type, ECM component name, mean, sample size, and standard deviation/standard error were extracted, together with information on subject age, sex, pathology, sub-location within the tissue as well as the methodological information. If the data within a paper were stratified by a variable such as age, sex, or region within a given tissue the data were entered as separate datasets. The complete data pool is available in Supplemental Table 1.

### Data transformation

The data that were not presented in the form of μg protein/mg dry tissue were adjusted using the following assumptions and resources: (1) ECM proteins + glycosaminoglycans account for the entirety of the decellularized dry weight of cartilage^17^; (2) water content of tendon is 62.5%^18^; (3) water content of skeletal muscle is 75%^19^; (4) if publications provided direct measurements of water content, that information was used in lieu of reference values for transformations within that study; (5) molar weights were taken from genecards.org for moles to mass transformations.

### Data synthesis

Analysis was performed using MetaLab^20^. We assumed random effects model, and calculated individual dataset weights (*w*_*i*_) using inverse variance weighting:

1 $${w}_{i}=\frac{1}{se{({\theta }_{i})}^{2}+{\tau }^{2}}$$

Where *se*(*θ*_*i*_) were standard errors for individual datasets, and τ was an inter-study variance. When only standard deviations *se*(*θ*_*i*_) were provided, *se*(*θ*_*i*_) were calculated using dataset sample size as:

2 $$se({\theta }_{i})=\frac{sd({\theta }_{i})}{\sqrt{{n}_{i}}}$$

Inter-study variance (${\tau }^{2}$) was calculated using the DerSimonian and Laird method

3 $${\tau }^{2}=\frac{Q-(N-1)}{c}$$

Where N was the number of datasets, and Q statistics (Q) and concordance statistics (c) were calculated as follows:

4 $$Q=\sum _{i}(se{({\theta }_{i})}^{-2}{({\theta }_{i}-\frac{{\sum }_{i}se{({\theta }_{i})}^{-2}{\theta }_{i}}{{\sum }_{i}se{({\theta }_{i})}^{-2}})}^{2})$$

5 $$c=\sum _{i}se{({\theta }_{i})}^{-2}-\frac{{\sum }_{i}{(se{({\theta }_{i})}^{-2})}^{2}}{{\sum }_{i}se{({\theta }_{i})}^{-2}}$$

Outcomes reported in individual datasets (${\theta }_{i}$) were synthesized into a weighted outcome ($\hat{\theta }$), according to individual dataset weights (${w}_{i}$):

6 $$\hat{\theta }=\frac{{\sum }_{i}({\theta }_{i}\cdot {w}_{i})}{{\sum }_{i}({w}_{i})}$$

95% confidence intervals (CI) were calculated using a Z distribution:

7 $$\pm CI=\pm \,1.96\cdot se(\hat{\theta }).$$

### Heterogeneity assessment

We report heterogeneity measures H^2^ and I^2^, which are in turn dependent on the total variation within our overall data pool (Q_total_):

8 $${Q}_{total}=\sum _{i=1}^{N}({w}_{i}\cdot {({\theta }_{i}-{\hat{\theta }}_{FE})}^{2})$$

where

$${\hat{\theta }}_{FE}=\frac{{\sum }_{i}se{({\theta }_{i})}^{-2}{\theta }_{i}}{{\sum }_{i}se{({\theta }_{i})}^{-2}}\,{\rm{a}}{\rm{n}}{\rm{d}}\,{w}_{i}=se{({\theta }_{i})}^{-2}$$

9 $${H}^{2}=\frac{{Q}_{total}}{df}$$

10 $${I}^{2}=\frac{{H}^{2}-1}{{H}^{2}}\cdot 100 \% .$$

### Cumulative and single-study exclusion plots

Cumulative exclusion plots were generated by iteratively removing the largest contributors to overall heterogeneity until a predefined homogeneity threshold was reached, assessed using a X^2^ distribution (p = 0.05). Single study exclusion plots assessed the effect of removal of each individual dataset. It is important to note that both cumulative and single study exclusion plots were constructed for the analysis of heterogeneity only, and the studies remained incorporated into our overall estimates.

### Funnel plots

Study level effects (*θ*_*i*_) were plotted in relation to their inverse standard error. Theoretical 95% CIs were included to assist in visualizing an unbiased dataset.

### Native tissue estimates

The estimates were generated for all tissues where 1) relative proteomic information was available; 2) an estimate for absolute amount of total collagen was calculated; 3) total collagen was included in the relative proteomic information. The estimated mass of a protein (*M*_*p*_) was calculated from the ratio of its relative amount (*R*_*p*_) to the relative amount of collagen (*R*_*Collagen*_) and the absolute estimate for the mass of collagen in the tissue (*R*_*Collagen*_)

11 $${M}_{p}=\frac{{R}_{p}}{{R}_{Collagen}}\,\times {M}_{collagen}.$$

When the estimates for non-collagenous tissue constituents calculated during the study were based on at least 3 datasets, they were used in lieu of (11).

## Results

### Study selection

**Figure 1:**
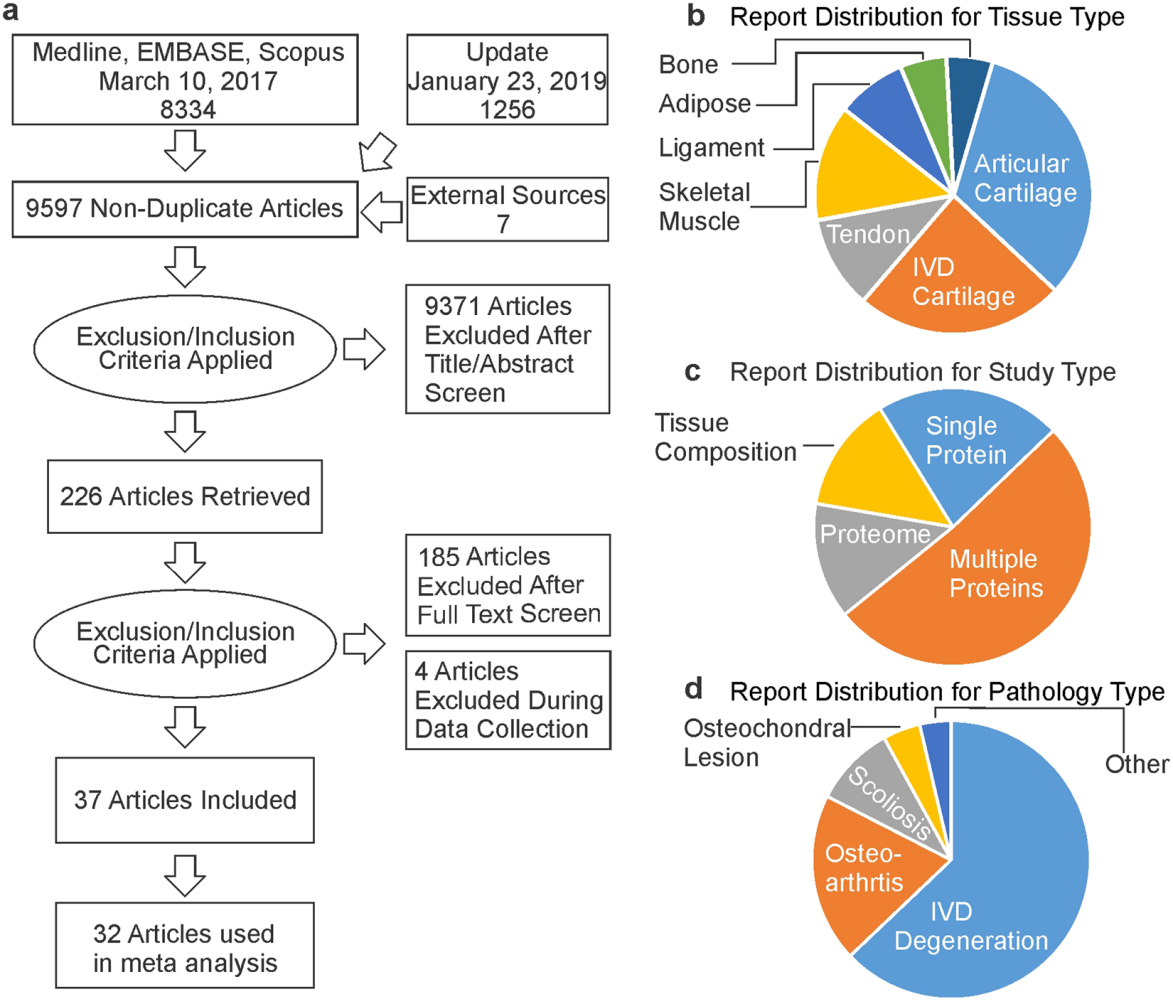
Search strategy and outcomes. (**a**) Prisma diagram for the information flow. **(b–d**) The distribution of information within 32 accepted papers concerning (**b**) tissue type; (**c**) the number of components reported within each study; and (**d**) pathology type.

The electronic search for reports containing quantification of ECM components in connective tissues identified 9,597 papers, of which 226 were included as potentially relevant after title/abstract screening. Full text screening yielded 37 articles containing quantitative information on at least one ECM protein. (Fig. 1a). From the selected articles, 12 reported the absolute quantitative information for proteins derived from articular cartilage^21–32^; 9 for intervertebral discs (IVD)^16,33–40^; 3 each for tendon^41–43^ and skeletal muscle^44–46^; 2 for ligament^41,42^; and 1 for adipose tissue (Fig. 1b)^47^. A single protein was quantified in 8 studies; multiple components in 19 studies; 5 studies quantified the entire tissue proteome; and 5 reported on tissue-level composition (Fig. 1c). Absolute quantification was provided in 29 papers, while 8 studies reported relative values. In the studies reporting absolute protein quantification, several stratified data by age, sex, tissue sub-location and pathological state, which we extracted as 580 individual datasets. Pathology types covered in the selected papers included 173 datasets for the ECM composition in states of IVD degeneration; 54 for osteoarthritis; 26 for scoliosis; 12 for osteochondral lesions and 6 for other pathologies, including diabetes and obesity (Fig. 1d). In total, the absolute amount of 89 unique components was reported (Supplemental Table 2). After data appraisal, we selected to perform meta-analysis for 4 most commonly reported proteins, which included collagen, elastin, fibronectin and proteoglycans, in addition to glycosaminoglycan. Four studies, all reporting on articular cartilage, were excluded from meta-analysis since they did not provide information on these 5 components.

### Data distribution and heterogeneity

**Figure 2:**
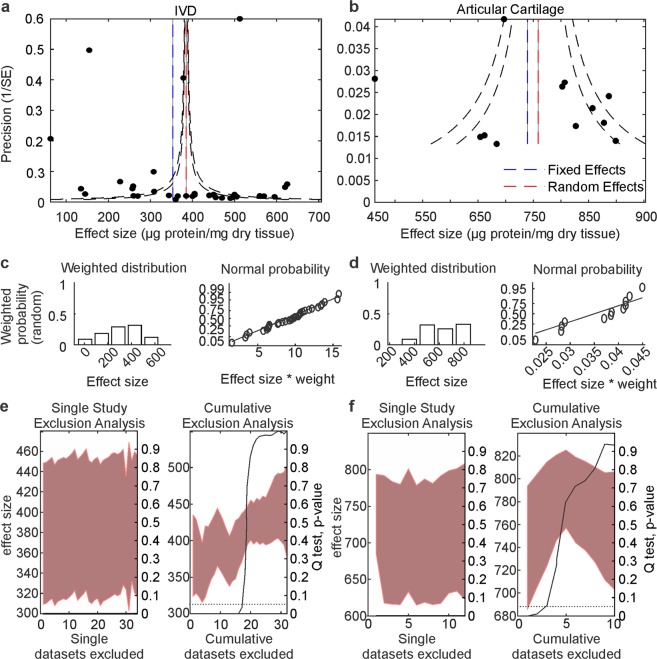
The data distribution and inter-dataset heterogeneity. (**a**,**b**) Funnel plots indicating bias and heterogeneity for collagen estimates in IVD (**a**) and articular cartilage (**b**). *Blue lines*: fixed effect model estimates, *red lines*: random effects model estimates; *black lines:* expected 95% confidence interval in the absence of bias/heterogeneity. (**c**,**d**) Histograms and normal probability plots for distribution of collagen estimates in IVD (**c**) and articular cartilage (**d**). (**e**,**f**) Single study (left) and cumulative (right) exclusion analysis for collagen estimates in IVD **(e)** and articular cartilage (**f**).

Collagen quantification in healthy IVD (34 datasets) and articular cartilage (12 datasets) were the largest data pools and thus most suited for synthesis and meta-analysis. We constructed funnel plots to investigate publication bias in the reported datasets, which would result in an asymmetric distribution of points on the plot about our total mean. The data points are distributed evenly about the estimated effect size for IVD (Fig. 2a), and for articular cartilage (Fig. 2b). The weighted distribution and normal probability plots indicated that values reported in the IVD data pool are approximately normally distributed (Fig. 2c), however the articular cartilage data deviated from normality (Fig. 2d). The single study and cumulative exclusion analysis (Fig. 2e) for the IVD data pool demonstrated that the individual study contributions to overall heterogeneity were fairly consistent, and removal of 53% of the datasets generated a homogenous data pool. In the articular cartilage data pool, no individual study markedly contributed to the overall heterogeneity, and removal of 29% of the datasets generated a homogenous data pool (Fig. 2f).

### Meta-analysis of collagen abundance in connective tissues

**Figure 3:**
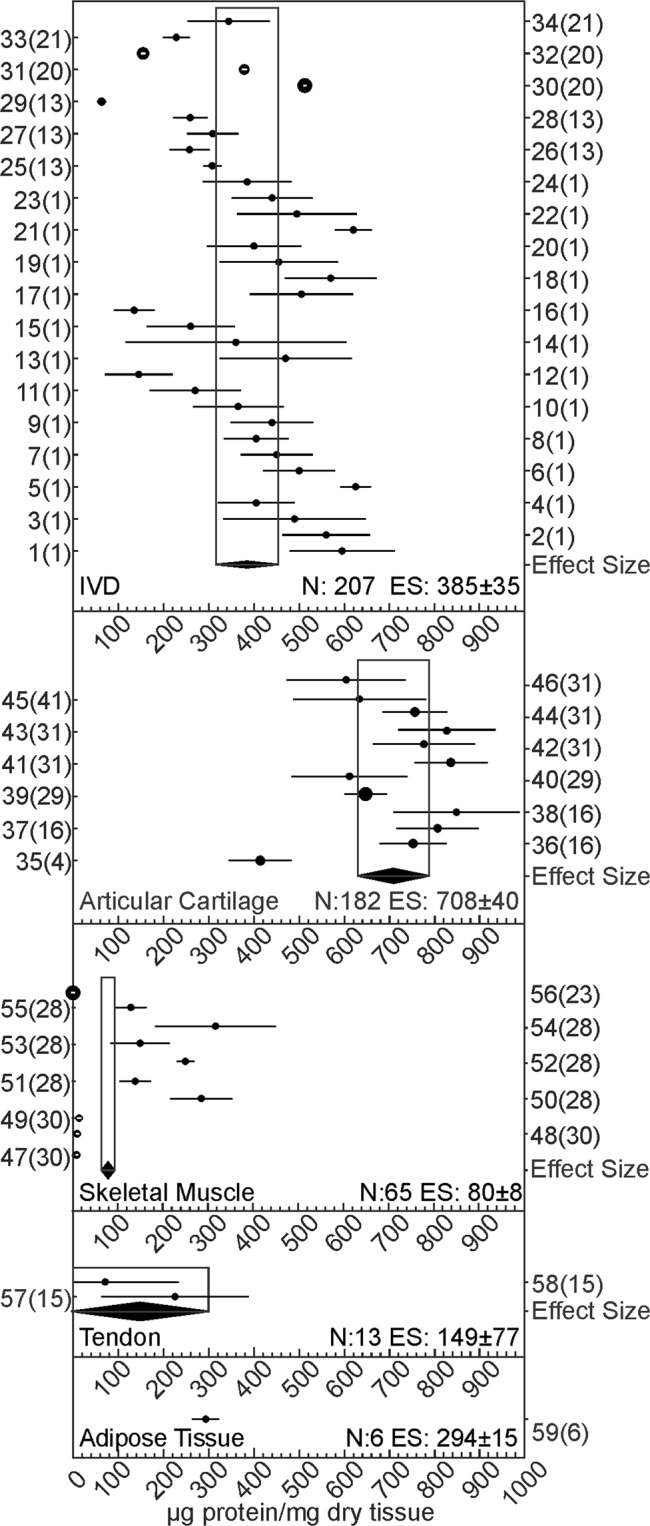
Collagen abundance in connective tissue. Forest plot for the estimated collagen abundance in IVD, articular cartilage, skeletal muscle, tendon, and adipose tissue. Shown are the effect sizes (black dots) with 95% CI (black lines) for each dataset included in the analysis and the overall effect sizes (ES) with 95% CI (black diamonds) for each tissue type. Y-axis labels indicate the included datasets and study numbers in the format dataset(study) according to Supplemental Table 1, where extended information can be found. The size of the circle is proportional to the number of datasets.

Using a random effects model, we estimated collagen abundance in connective tissues (Fig. 3). IVD (n = 34 datasets reporting N = 207 samples) was found to contain 385 μg collagen/mg dry tissue (95% confidence interval (CI): 350, 420); articular cartilage (n = 12 datasets reporting N = 182 samples) – 708 μg collagen/mg dry tissue (95% CI: 668, 748), skeletal muscle (n = 10 datasets reporting N = 65 samples) – 80 μg collagen/mg dry tissue (95% CI: 72, 88), and tendon (n = 2 datasets reporting N = 13 samples) – 149 μg collagen/mg dry tissue (95% CI: 72, 226). We identified a single dataset for adipose tissue (6 samples) which reported collagen abundance to be 294 μg collagen/mg dry tissue (95% CI: of 279, 309). Two cartilaginous tissues, IVD and articular cartilage, were found to contain significantly different amounts of collagen.

### Investigation of methodological and biological contributors to heterogeneity

**Figure 4:**
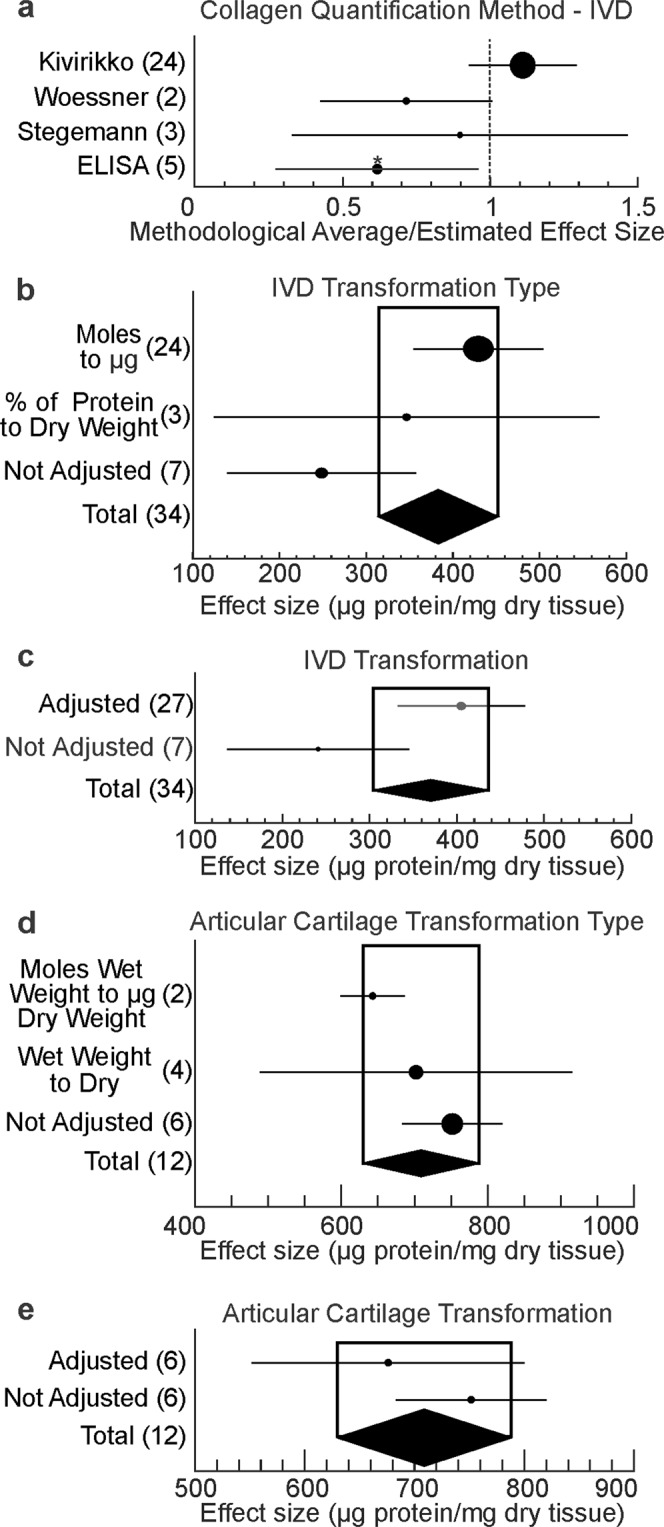
Technical contributors to inter-study differences. Forest plots for the estimated effect size of collagen abundance for different quantification methods (**a**), and original data transformations (**b–e**). For **a**, data are presented as a ratio to the overall estimate. For (**b**–**e**), shown are IVD (**b**,**c**) and articular cartilage (**d**,**e**) subgroup averages and 95% CI as black dots/black lines for different type of transformations (**b**,**d**), or adjustment (**c**,**e**). The overall effect sizes and 95% CI as black diamonds. The size of the circle is proportional to the number of datasets, *indicates statistical significance.

**Figure 5:**
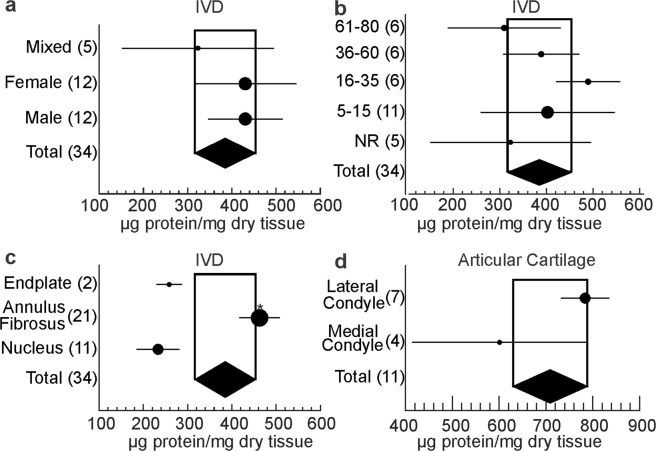
Estimating the contribution of biological factors to collagen abundance in IVD and articular cartilage. Forest plot for the estimated collagen abundance in IVD **(a–c)** and articular cartilage (**d**) as a function of sex (**a**), age (**b**), and tissue sub-location (**c**,**d**). Shown are subgroup averages and 95% CI as black dots/black lines, the overall effect sizes and 95% CI as black diamond, the number of datasets included within each subgroup is indicated in parenthesis. The size of the circle is proportional to the number of datasets; *indicates statistical significance.

Since the degree of heterogeneity in the two most populated cartilage data pools was high (IVD: H^2^ = 708, I^2^ = 99.9%; articular cartilage: H^2^ = 9.6, I^2^ = 89.6%), we next investigated whether factors related to methodological choices (Fig. 4) or biological factors (Fig. 5) contributed to the heterogeneity of the datasets. Different methodologies were employed to quantify collagen levels, including original hydroxyproline quantification using Stegemann technique (3 datasets) and its Kivirikko (24 datasets) and Woessner (2 datasets) modifications, as well as ELISA (5 datasets). Stratification of the data demonstrated that significantly lower collagen content was estimated using ELISA compared to other techniques (Fig. 4a). Studies reported the protein abundance in moles, micrograms, or a percent of total protein in relation to the wet or dry weight of tissue. Therefore, the dataset values were transformed to micrograms per milligram of dry tissue weight prior to meta-analysis. We examined if transformation of the data systematically affected the outcome in IVD (Fig. 4b,c) and articular cartilage (Fig. 4d,e) data pool, however data transformation did not significantly contribute to differences in outcome.

Several biological variables that could contribute to cartilage ECM composition were reported, including subject sex, age, and tissue sub-location. Subject sex or age did not significantly affect collagen abundance in IVD, even though an interesting age-dependent change was visually evident (not statistically significant by ANOVA, p = 0.25) (Fig. 5a,b). In IVD collagen abundance was significantly higher in the annulus fibrosus relative to both the endplate and the nucleus pulposus (Fig. 5c). Heterogeneity was reduced and data pools exhibited low bias and normal distribution (Fig. S1) when IVD data pools for annulus fibrosus (n = 4 reporting N = 113 samples, H^2^ = 124.4, I^2^ = 99.2%) and nucleus pulposus (n = 4 reporting N = 60 samples, H^2^ = 43.5, I^2^ = 97.7%) were separated. Collagen abundance in the lateral and medial condyle of articular knee cartilage was similar, however large variability in medial condyle reports was evident (Fig. 5d).

### Estimated abundance for sGAG, fibronectin, elastin and proteoglycans

Robust analysis for non-collagenous ECM components was constrained by limited numbers of reports. Nevertheless, we estimated the content of sGAG, fibronectin, elastin, and proteoglycans in tendon, skeletal muscle, articular cartilage, ligament, and IVD (Table 1).

**Table 1:** The estimated abundance of sulfated glycosaminoglycans (sGAG), fibronectin, elastin, and total proteoglycans in indicated tissues. For each component/tissue, n is the number datasets that reported in total N samples.

Component	Tissue	n	N	Amount (μg/mg dry tissue)	CI
sGAG	Tendon	2	13	34	±57
	Skeletal Muscle	6	40	196	±22
	Articular Cartilage	8	158	56	±23
Fibronectin	Tendon	2	6	1.6	±0.3
	Ligament	2	6	11	±7.0
	Articular Cartilage	2	5	2.3	±2.6
Elastin	Tendon	3	16	186	±300
	IVD Cartilage	3	24	18	±4.1
Proteoglycans	IVD Cartilage	2	20	144	±16
	Articular Cartilage	4	25	120	±46

### Changes in ECM composition in pathological samples

**Figure 6:**
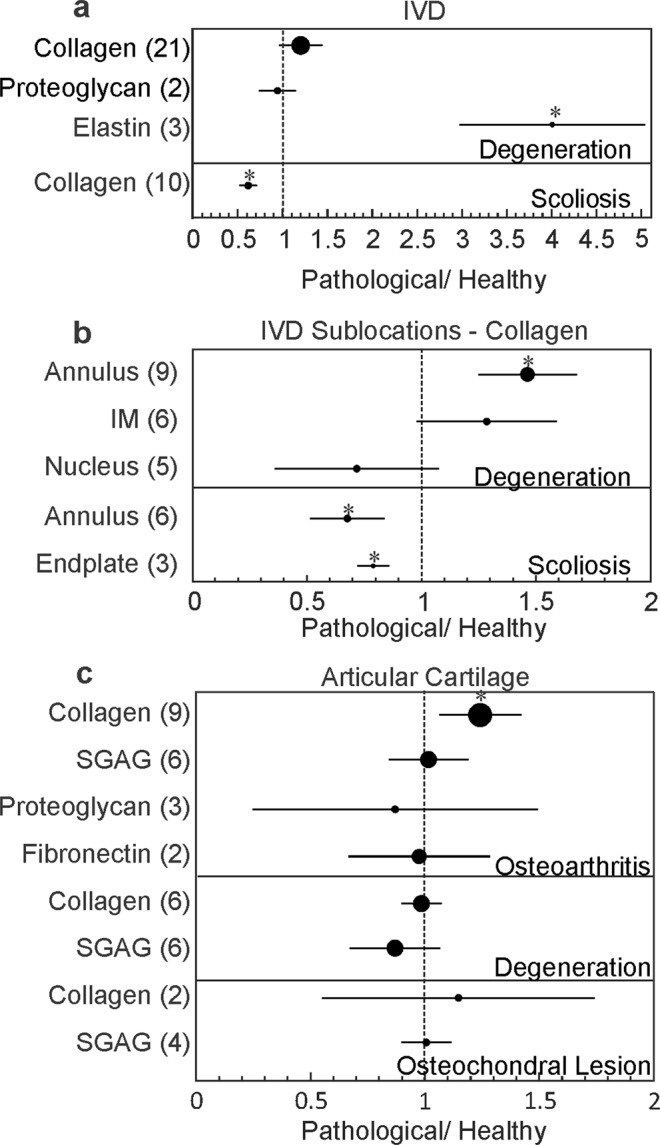
Forest plot for the estimated effect of pathologies on the abundance of ECM components. (**a**,**b**) Effect of IVD degeneration and scoliosis on abundance of collagen, proteoglycans and elastin in IVD (**a**) and collagen content in IVD sub-locations (**b**). (**c**) Effect of osteoarthritis, cartilage degeneration and osteochondral lesion on abundance of collagen, sGAG, proteoglycans and fibronectin in articular cartilage. Shown are the estimated effect sizes (black circles) and 95% CI (black lines) for the abundance of indicated components in pathological tissues relative to healthy estimates (1 indicates equal amounts, dashed vertical line); the number of datasets included within each group is indicated in parenthesis. The size of the circle is proportional to the number of datasets, *indicates statistical significance.

ECM composition in a pathological state was reported in 18 studies (Table 2). Disc degeneration was associated with a significant increase in elastin abundance, while scoliosis resulted in a significant decrease in collagen (Fig. 6a). Since we had identified IVD location as a significant determinant of collagen content, we further investigated if IVD pathologies differentially affect collagen content in different parts of IVD. We have found that IVD degeneration resulted in a significant increase in collagen in annulus fibrosus, but not in nucleus pulposus or intermediate zone (Fig. 6b). Scoliosis was associated with a reduction in collagen content in annulus fibrosus and in the endplate (Fig. 6b). In osteoarthritic articular cartilage, the collagen content was significantly increased, but articular cartilage degeneration or osteochondral lesions were not associated with changes in collagen or sGAG (Fig. 6c).

**Table 2:** The estimated effects of various pathologies (osteoarthritis, IVD degeneration, scoliosis, osteochondral lesion, obesity, diabetes) on the abundance of indicated components in pathological tissues relative to healthy estimates (1 indicates equal amounts).

Component	Tissue	Pathology	n	N	Pathological / Healthy	CI
Collagen	IVD	Degeneration	21	291	0.79	±0.25
		Scoliosis*	10	147	0.51	±0.08
	IVD Annulus	Degeneration*	9	116	1.5	±0.20
		Scoliosis*	6	132	0.67	±0.16
	IVD Intermediate Zone	Degeneration	6	98	1.3	±0.30
	IVD Nucleus	Degeneration	5	63	0.72	±0.36
	IVD Endplate	Scoliosis*	3	45	0.54	±0.04
	Articular Cartilage	Osteoarthritis*	9	91	1.24	±0.18
		Degeneration	6	72	0.98	±0.09
		Osteochondral Lesion	2	17	1.1	±0.6
	Skeletal Muscle	Diabetes	1	10	2.2	±1.4
		Obesity	1	10	2.1	±1.3
sGAG	Articular Cartilage	Osteoarthritis	6	78	1.06	±0.16
		Degeneration	6	72	0.95	±0.27
		Osteochondral Lesion	4	64	1.13	±0.24
Fibronectin	Articular Cartilage	Osteoarthritis	2	13	0.99	±0.16
Elastin	IVD	Degeneration*	3	15	4.0	±1.0
Proteoglycans	IVD	Degeneration	2	17	0.94	±0.20
	Articular Cartilage	Osteoarthritis	3	20	0.87	±0.63

For each component/tissue/pathology, n is the number datasets that reported in total N samples. *Indicates significant difference from physiological values.

### Overall composition of connective tissues

**Figure 7:**
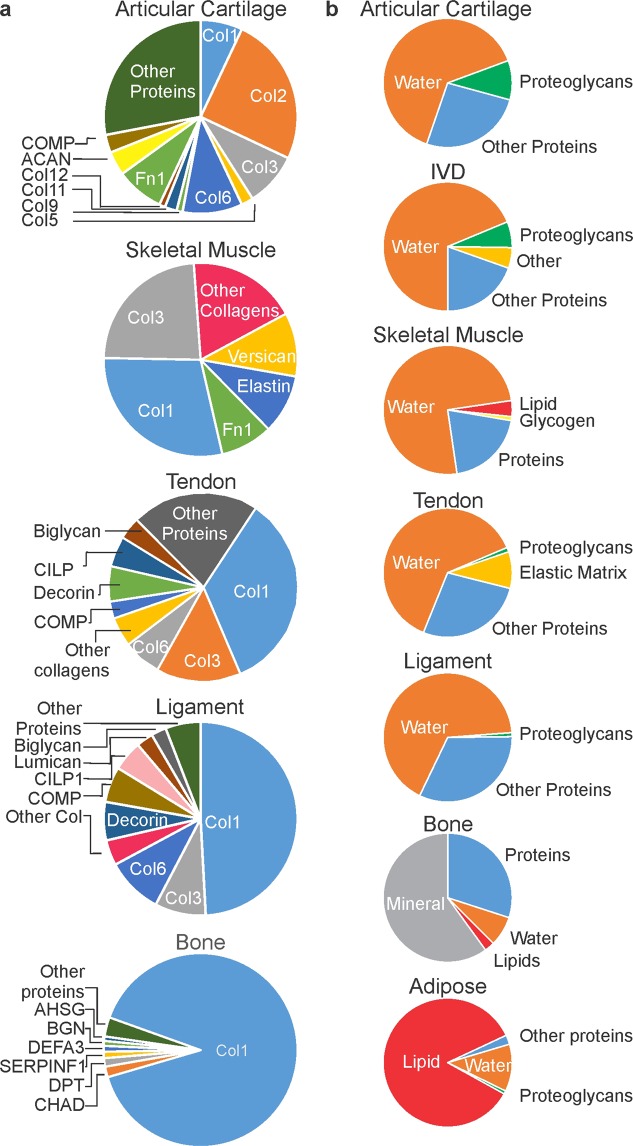
Comparative proteomic and overall composition of different connective tissues. (**a**) Relative abundance of ECM proteins derived from studies that investigated the large numbers of proteins or complete proteomes in articular cartilage, skeletal muscle, tendon, ligament, and bone. For articular cartilage, skeletal muscle, ligament, and bone the data are from a single study; for tendon, relative abundance of top 10 proteins identified by both studies was averaged. (**b**) Relative quantity of whole tissue constituents in articular cartilage, skeletal muscle, ligament, tendon, bone, adipose tissue, and IVD.

Some of the selected studies reported the proteomic analysis of tissue samples. While these studies do not provide absolute quantification of the identified proteins, we compiled the relative quantification data to obtain an overall portrait of protein composition of different connective tissues. The relative makeup of the proteomes of articular cartilage^25^, skeletal muscle^48^, tendon^42,49^, ligament^42^, and bone^50^ were reported (Fig. 7a). To determine the relative composition of the whole tissues, we combined the proteomics data with the estimated amounts of water or unique constituents such as lipids, or mineral for articular cartilage^17,30^, IVD^16,51^, skeletal muscle^19^, tendon^18^, ligament^52^, bone^53^, and adipose tissue^54^ (Fig. 7b). Finally, combining the relative quantifications with calculated estimates of absolute abundance of collagens, we calculated the estimated levels for all tissue constituents in articular cartilage **(**Table 3**)** skeletal muscle **(**Table 4**)**, and tendon (Table 5).

**Table 3:** Estimated whole-tissue composition of articular cartilage.

Component	Amount (ug/mg articular cartilage)	±
Collagen 1	23.0	2.6
Collagen 2	82.1	9.2
Collagen 3	29.6	3.3
Collagen 5	6.57	0.73
Collagen 6	32.8	3.7
Collagen 9	3.28	0.37
Collagen 11	6.57	0.73
Collagen 12	3.28	0.37
Total Collagens	187	21
Fibronectin	26.3	2.9
COMP	9.9	1.1
Other Proteins	92	10
Total	315	35
Water	650	
Aggrecan	49.8	5.6
Other Proteoglycans	70	
Total Proteoglycans	120	46
Total	1085	82

**Table 4:** Estimated whole-tissue composition of skeletal muscle.

Component	Amount (μg/mg skeletal muscle)	±
Collagen 1	32.6	6.0
Collagen 3	26.6	4.9
Other Collagens	20.6	3.8
Total Collagens	80	15
Versican	12.0	2.2
Elastin	11.1	2.1
Fibronectin	9.9	1.8
Total Proteins	113	21
Water	750	20
Lipid	40	
Glycogen	10	
Total	913	41

**Table 5:** Estimated whole-tissue composition of tendon.

Component	Amount (μg/mg tendon)	±
Collagen 1	103	38
Collagen 3	17.8	6.6
Collagen 6	19.8	7.3
Other Collagens	8.9	3.3
Total Collagens	149	77
COMP	12.4	4.6
CILP1	10.5	3.9
Other Proteins	12.3	4.5
Total Proteins	184	90
Decorin	13.4	4.9
Lumican	5.9	2.2
Biglycan	5.2	1.9
Total Proteoglycans	24.5	9.0
Water	625	
Other (elastic matrix)	92	
Total	926	99

## Discussion

In this paper we synthesized the existing literature and generated robust estimates for collagen content in human connective tissues. In addition, within the IVD, we quantified the regional collagen content in the annulus fibrosus, nucleus pulposus, and endplate. Analysis of methodological techniques identified systematic differences between the estimates provided by ELISA and by the hydroxyproline-based assays. Collagen abundance was not significantly affected by sex or age. We demonstrated that osteoarthritis, disk degeneration and scoliosis lead to distinct changes in ECM composition, particularly in the abundance of collagen and elastin. Finally, we synthesized known information on absolute and relative protein content, as well as water, polysaccharide and unique tissue components such as lipid, to provided quantitative estimates for the native composition of human connective tissues.

Our primary goal was to synthesize existing research to build robust estimates of ECM component abundance. This question may appear outdated, as many publications and reviews present certain facts about tissue composition as common knowledge, such as cartilage containing somewhere between 20 and 35% protein, with the majority of that fraction being collagen and proteoglycans^17,55^. However, it is very difficult to follow the citation trail to find the origins of these estimates. We performed a systematic review in order to identify the primary papers reporting direct quantification of ECM compositions in human connective tissues. Based on these studies we were able to perform meta-analysis on a limited number of tissues and components. Importantly, for some tissues, such as bone, no study has passed the inclusion criteria, while for other tissues, such as adipose tissue and ligament, only 1–2 studies were identified, demonstrating significant gaps in information regarding quantification of absolute amounts of ECM components in human tissues.

The datasets describing collagen quantification in articular and IVD cartilage were sufficiently large and of high enough quality to obtain robust estimates and perform more in depth meta-analysis. While heterogeneity was high, which may negatively affect the precision of the estimates, the majority of the datasets for both IVD and articular cartilage data pools were evenly distributed about the random effects estimate within the expected 95% CI. No single study was found to account for a significant portion of overall heterogeneity. Methodologically, collagen abundance was assessed by measuring hydroxyproline content using Stegemann, Kivirikko, or Woessner methods^56–58^ in 29 of 35 studies or by ELISA in 6 studies. The mean of the estimates generated by ELISA were significantly lower than the overall estimate, suggesting that systematic methodological differences need further investigation. We have found that the sex or age of the subjects did not significantly affect IVD collagen abundance. It is known that articular cartilage contains proportionally more total protein than IVD^17,51^. Our estimates suggest that, in addition, collagen accounts for different proportions of total protein in these two tissues. Within the IVD we demonstrated that collagen abundance differs between the annulus fibrosus and nucleus pulposus, with the annulus having significantly more collagen. This is consistent with the current literature, which asserts that the dry-weight of the nucleus is shifted towards proteoglycans^59^. Importantly, pathological conditions were associated with significant and disease-specific changes in cartilage collagen content. Degeneration of IVD was associated with a significant increase in collagen abundance in the annulus fibrosus. Of interest, collagen content in nucleus pulposus of degenerating disks tended to decrease, providing biochemical basis for location-specific changes in degenerating IVD, where altered collagen distribution was proposed to contribute to the loss of structural integrity and horizontal bulging of the disc^59^. Similar to disk degeneration, the articular cartilage of osteoarthritis patients was found to contain more collagen than healthy tissue. Since increased deposition of ECM proteins was previously linked to fibrosis and injury repair of cartilage^60^, high collagen content in osteoarthritic and degenerating cartilage may be related to its repair and regeneration. Tears in degrading discs tend to occur in the annulus of IVD, consistent with higher collagen content in the annulus rather than nucleus of degenerating disks^59^. In contrast to cartilage degeneration, scoliosis was associated with a reduction in collagen abundance, in particular in the annulus and the endplate, supporting the theory that abnormalities in the IVD ECM are involved in the pathophysiology of scoliosis^61^. Thus, although individual studies reported heterogeneous estimates, given sufficient number of primary studies, the data still can be successfully synthesized to provide significant insights into the physiology and pathology of connective tissues.

In contrast to collagen, reports on other ECM components were much less common, which limited data potential for synthesis. We built estimates for the abundance of sGAG in tendon, skeletal muscle, and articular cartilage; fibronectin in tendon, ligament, and articular cartilage; elastin in tendon and cartilage; and total proteoglycans in IVD and articular cartilage. Limited number of reports prevented further analysis of these data. Our results suggest that the abundance of these components can be affected in pathological conditions, such as an increase in elastin in the degenerating disc. Thus, it is important to further investigate the abundance of non-collagenous ECM components, how they vary between tissues, and how they change in disease.

We generated relative protein breakdowns for 5 tissues (articular cartilage, skeletal muscle, tendon, ligament, and bone) and relative component proportions for 7 tissues (articular cartilage, IVD, skeletal muscle, tendon, ligament, bone, and adipose tissue). For three tissues, articular cartilage, skeletal muscle and tendon, we had an absolute estimate for the total collagen content, a proteomic breakdown that included collagen, and the estimates for other components, allowing us to generate numerical values accounting for 100% of the native tissue (1000 μg constituents/1 mg wet tissue). The summation of our individual estimates for articular cartilage (1085 ± 82) was slightly larger than expected. Our overall estimate for skeletal muscle (913 ± 41) was slightly lower than expected. Our 95% confidence interval for tendon (926 ± 99) includes 1000 μg constituents/1 mg tissue mass. The small amount of deviation from expected values in articular cartilage could result from systematic overestimation of collagen content by hydroxyproline based quantification methods. In regards to skeletal muscle, the reference proteomic study was not aimed at describing the complete proteome, and is missing some primary constituents of skeletal muscle such as its most abundant proteoglycan, decorin^62^. Proteins and proteoglycans are thought to make up 20% of skeletal muscle, while we only account for 11.2%. Incorporation of the remaining ~9%, translating to 90 μg, would put our overall calculation right around 1000 μg constituents/1 mg tissue. An additional uncertainty is due to our use of static reference values for total water content, which likely fails to take into consideration individual subject variations. Overall, the fact that for these 3 tissues the overall numerical estimates very closely (within 0.3–17%) account for an expected tissue mass strongly attests to the validity of underlying assumptions and calculations.

Outside of the scope of our findings themselves, academia is facing a systematic problem whereby flawed experimental design and selective reporting give rise to data that are erroneous, irreproducible, or cherry-picked^63^. The synthesis of published studies is often used in the context of clinical trial evaluation and is considered to be the gold standard of evidence^64^. In principle, synthesis can also be used to generate more robust and high powered estimates for virtually any quantitative scientific question, however the technique is seldom used in basic research^20^. The generated estimates effectively employ a much larger sample size than any single study, and through analysis of the datasets themselves, researchers can identify systematic reporting biases and contributors to inter-study variations. It is in the interest of all researchers to use these tools to cost-effectively improve upon the existing bank of knowledge. It is important to note that the quality of these estimates, and thus the effectiveness of the technique, strongly depends on the quality of the underlying publications. In our case, absolute quantification was not available for many tissues or for proteins other than collagen. Relative quantification using proteomics techniques has been performed for the number of tissues, however transforming relative quantification data from mass spectrometry to absolute estimates is difficult, and these studies were rarely reproduced. In therapy development, limitations in modern pre-clinical disease models contribute to a failure to translate *in-vitro* success to clinical trial success^63^. It is well understood that the physical properties of the ECM, in addition to the proteins themselves, are powerful regulators of cellular behavior. Thus, failure to adequately replicate the native extracellular environment when culturing cells *in vitro* may lead to their altered behavior, and as a result, what is observed may not translate into the human patient^65^. Culturing on ECM-coated dishes, 3D culture on biomimetic scaffolds, and culture of organ-like organoids has become more and more commonplace^65,66^. By building a quantitative ingredient list for the extracellular environment we will be able to refine and evaluate these models and further improve upon our repertoire of *in vitro* tools.

## Supplementary information

Supplemental materials

Supplemental table 1
