# Supplementary material for: Extracellular matrix composition of connective tissues: a systematic review and meta-analysis
Source: Sci Rep. 2019 Jul 22;9:10542. doi: 10.1038/s41598-019-46896-0 (PMC6646303; doi:10.1038/s41598-019-46896-0)
Supplement: Supplementary file 1 — Supplemental materials [file 41598_2019_46896_MOESM1_ESM.pdf]

Supporting material for the manuscript “Extracellular matrix composition of connective tissues: systematic review and meta-analysis” by T. McKee, G. Perlman, M. Morris, and S.V. Komarova.

Supplemental Text 1. Search strategy for identifying articles.

1. Extracellular Matrix/
2. Microfibrils/
3. (extracellular matrix or ECM).ti,ab,kf.
4. 1 or 2 or 3
5. exp Extracellular Matrix Proteins/
6. (alcam or activated-leucocyte or (cd166 adj1 (antigen* or ligand*)) or kg-cam or neurolin or ADAMTS* or adam metallopeptidase? with thrombospondin or aggrecan? or cartilage-specific proteoglycan core or connective tissue growth factor or hypertrophic chondrocyte specific protein 24 or insulin like growth factor binding protein 8 or cysteine-! rich protein 61 or collagen? or procollagen? or tropocollagen? or endostatin? or elastin? or tropoelastin? or fibrillin* or fibronectin or cold-insoluble globulin? or (sialoprotein adj3 (bone or integrin binding)) or laminin or merosin or matrilin or osteopontin or tenascin* or cytotactin or hexabrachion or j1-200-220 or versican? or vitronectin or ((sirp or small leucine rich) adj3 proteoclycan?) or ((alpha 2 surface binding or gp 2 or matrix or opsonic) adj3 glycoprotein) or ((cartilege oligomeric matrix or cyr ctgf nov matricellular or (ccn adj3 (intercellular signal?ing or family or matricellular)) or thrombospondin 5 or ccn1 or ccn3 or cyr61 or igfbp-8 or igfbp-rp2 or igfbp9 or novh or igfbp10 or matn? or nephroblastoma overexpressed or latent tgf beta binding or cartilage matrix or igf binding or insulin-like growth factor binding or (ECM adj5 regulat*)) adj3 protein?)).ti,ab,kf.
7. 5 or 6
8. exp Adipose Tissue/
9. Myocardium/ or Myocytes, Cardiac/
10. exp Muscle, Skeletal/
11. Endothelium, Vascular/
12. exp "Bone and Bones"/
13. exp Cartilage/
14. (((cardiac or skeletal) adj1 muscle?) or bone? or adipose tissue or myocardium or vascular endotheli* or cartilage).ti,ab,kf.
15. or/8-14
16. 4 and 7 and 15
17. (characteri?e or assess or composition* or analy?e or analysis or isolate? or isolation or differentiat* or quantif* or characteri* or quantitativ* or proteomic* or protein content* or qconcat or component*).ti,ab,kf.
18. exp Proteomics/
19. 17 or 18
20. (decellulari* or acellular*).ti,ab,kf.
21. Extracellular Matrix/ph
22. Microfibrils/ph
23. 21 or 22
24. 4 and 7 and 15 and 19
25. 23 and 15 and 19
26. 4 and 15 and 20
27. or/24-26
28. limit 27 to animals/
29. limit 27 to humans/
30. 28 not (28 and 29)
31. 27 not 30

Supplemental Table 2. List of proteins reported in 32 included articles that contained absolute quantification. Shown are tissues in which the protein was reported, the number of times each protein was reported on in healthy and pathological conditions and corresponding references. References correspond to the paper legend available in supplementary table 1.

	Count Healthy	Count Pathological	Tissue Type	Ref
a1 Antitrypsin	4	0	3;7	14
Actin - Cytoplasmic 1/2	1	0	4	10
Actin - Total	1	0	4	10
Aggrecan	1	2	2	7;8
Agrin	1	0	4	10
Agrin (iso 2,3,4,5,&6)	1	0	4	10
Albumin	4	0	3;7	14
Alpha-1 Microglobulin/Bikunin Precursor	4	0	3;7	14
Annexin A2	1	0	4	10
Apolipoprotein A1	2	0	3	14
Apolipoprotein A4	2	0	3	14
Apolipoprotein E	2	0	3	14
asporin	4	0	3;7	14
Biglycan	1	0	4	10
Cartilage Intermediate Layer Protein	1	2	2	4
Cartilage Oligomeric Matrix Protein	2	4	2	4;7;8
Chondroadherin	2	0	3	14
Chondroitin Sulphate (846 Epitope)	5	43	2;8	12;13;24;29
CSPG Core Protein 2	2	0	3	14
Clusterin	4	0	3;7	14
Collagen (Col12A1)	5	0	3;4;7	10;14
Collagen (Col18A1)	1	0	4	10
Collagen (Col1A1)	5	0	3;4;7	10;14
Collagen (Col1A2)	5	0	3;4;7	10;14
Collagen (Col3A1)	4	0	3;7	14
Collagen (Col4A1)	1	0	4	10
Collagen (Col4A1/5)	1	0	4	10
Collagen (Col4A2)	1	0	4	10
Collagen (Col4A5)	1	0	4	10
Collagen (Col5A1)	1	0	4	10
Collagen (Col5A2)	1	0	4	10
Collagen (Col6A1)	5	0	3;4;7	10;14
Collagen (Col6A2)	5	0	3;4;7	10;14

Collagen (Col6A3)	5	0	3;4;7	10;14
Collagen (Insoluble)	1	2	4	19
Collagen (Soluble)	1	2	4	19
Collagen (total)	59	51	1;2;5;7;8	1;4;6;11;12;13;15;16;20;21;22;23;24;28;29;30;31
Collagen (type 1)	3	0	2;3;7	7;18
Collagen (type 11)	1	0	2	7
Collagen (type 12)	1	0	2	7
Collagen (type 2)	3	18	2;3;7;8	7;12;17;18
Collagen (type 3)	1	0	2	7
Collagen (type 5)	1	0	2	7
Collagen (type 6)	1	0	2	7
Collagen (type 9)	1	0	2	7
Collagen II Degradation Protein (C2C)	0	2	2	24
Complement Component 3	4	0	3;7	14
Complement Component 4 Binding Protein α	2	0	3	14
Complement Component 9	4	0	3;7	14
C-propeptide of type 1 procollagen	4	17	8	12;13
C-propeptide of type 2 procollagen	0	37	2;8	12;24
Decorin	1	0	4	10
Dermatopontin	1	0	4	10
Desmin	1	0	4	10
Elastin	9	5	1;3;4;7;8	6;15;18;19;20
Emilin-1	1	0	4	10
Fibrillin-1	1	0	4	10
Fibrinogen	2	0	3	14
Fibrinogen Beta	2	0	3	14
Fibrinogen Gamma	2	0	3	14
Fibromodulin	5	4	2;3;7	4;8;14
Fibronectin	8	4	2;3;4;7	4;5;7;10;14
Fibulin-5	1	0	4	10
Galactin-1	1	0	4	10
Laminin Gamma-1	1	0	4	10
Laminin Subunit Alpha-2	1	0	4	10
Laminin Subunit Alpha-5	1	0	4	10
Laminin Subunit Beta-1	1	0	4	10
Laminin Subunit Beta-2	1	0	4	10
LTBP-1	1	0	4	10

Lubricin (PRG4)	4	0	3;7	14
Lumican	5	0	3;4;7	10;14
Microfibrillar-associated protein 2	1	0	4	10
Mimecan/Osteoglycin	5	0	3;4;7	10;14
MMP-1	0	2	2	8
MMP2 (Pro-MMP2)	0	1	8	11
MMP-3	0	2	2	8
Myosin (Myosin-3,4,6,7)	2	0	4	10
Nidogen-1	1	0	4	10
Nidogen-2	1	0	4	10
Pentraxin-2 (SAMP)	4	0	3;7	14
Periostin	2	1	2;4	2;10
Perlecan - HSPG2	5	0	3;4;7	10;14
Phosphatidylglycerophosphate Synthase 1	4	0	3;7	14
Phosphatidylglycerophosphate Synthase 2	4	0	3;7	14
Phosphoglucomutase	2	0	3	14
Plasminogen	0	2	2	8
Prolargin (PRELP)	6	2	2;3;4;7	4;10;14
Protein (Total)	1	1	8	13
Proteoglycan (total)	7	6	2;8	4;16;21;24;26
Sulphated Glycosaminoglycan	19	59	1;2;4;5;7;8	6;9;12;15;17;19;22; 28;29;31
Tenascin C	1	1	2	25
TIMP-1	0	3	2;8	8;11
TnxB Protein	1	0	4	10
TGFβ-induced-protein ig-h3	5	0	3;4;7	10;14
Transglutaminase 2	1	0	4	10
Transthyretin	4	0	3;7	14
Tubulin beta	1	0	4	10
Vimentin	1	0	4	10
